# Factors associated with acute kidney injury in patients undergoing extracorporeal membrane oxygenation: retrospective cohort

**DOI:** 10.1590/1980-220X-REEUSP-2022-0299en

**Published:** 2023-04-17

**Authors:** Filipe Utuari de Andrade Coelho, Barbara Gadioli, Flavia Fernandes Manfredi de Freitas, Maria de Fatima Fernandes Vattimo

**Affiliations:** 1 Universidade de São Paulo, Escola de Enfermagem, São Paulo, SP, Brazil.; 2 Hospital Israelita Albert Einstein, Departamento de pacientes graves, São Paulo, SP, Brazil.

**Keywords:** Intensive Care Units, Extracorporeal Membrane Oxygenation, Acute Kidney Injury, Nursing, Unidades de Cuidados Intensivos, Oxigenación por Membrana Extracorpórea, Lesión Renal Aguda, Enfermería, Unidades de Terapia Intensiva, Oxigenação por Membrana Extracorpórea, Injúria Renal Aguda, Enfermagem

## Abstract

**Objective:**

To identify factors associated with acute kidney injury in patients undergoing extracorporeal membrane oxygenation.

**Method:**

Retrospective cohort study conducted in an adult Intensive Care Unit with patients undergoing extracorporeal membrane oxygenation from 2012 to 2021. The research used the Kidney Disease Improving Global Outcomes as criteria for definition and classification of acute kidney injury. A multiple logistic regression model was developed to analyze the associated factors.

**Results:**

The sample was composed of 122 individuals, of these, 98 developed acute kidney injury (80.3%). In multiple regression, the associated factors found were vasopressin use, Nursing Activities Score, and glomerular filtration rate.

**Conclusion:**

The use of vasopressin, the Nursing Activities Score, and the glomerular filtration rate were considered as factors related to the development of acute kidney injury in patients undergoing extracorporeal membrane oxygenation.

## RESUMO

**Objetivo:**

Identificar os fatores associados à lesão renal aguda em pacientes submetidos a oxigenação por membrana extracorpórea.

**Método:**

Estudo de coorte retrospectivo, realizado em uma Unidade de Terapia Intensiva adulta, com pacientes submetidos à oxigenação por membrana extracorpórea, no período de 2012 a 2021. O critério para definição e classificação da lesão renal aguda foi o da *Kidney Disease Improving Global Outcomes*. Para a análise dos fatores associados foi elaborado um modelo de regressão logística múltipla.

**Resultados:**

A amostra foi composta por 122 indivíduos, destes, 98 desenvolveram lesão renal aguda (80,3%). Na regressão múltipla, os fatores associados encontrados foram a utilização de vasopressina, o *Nursing Activities Score* e a taxa de filtração glomerular.

**Conclusão:**

O uso da vasopressina, o *Nursing Activities Score* e a taxa de filtração glomerular foram considerados como fatores relacionados ao desenvolvimento de lesão renal aguda em paciente submetido à oxigenação por membrana extracorpórea.

## RESUMEN

**Objetivo:**

Identificar los factores asociados a la lesión renal aguda en pacientes sometidos a oxigenación por membrana extracorpórea.

**Material y método:**

Estudio de cohortes retrospectivo realizado en una unidad de cuidados intensivos de adultos con pacientes sometidos a oxigenación por membrana extracorpórea entre 2012 y 2021. El criterio de definición y clasificación de lesión renal aguda fue el *Kidney Disease Improving Global Outcomes*. Se desarrolló un modelo de regresión logística múltiple para el análisis de los factores asociados.

**Resultados:**

La muestra estuvo compuesta por 122 individuos, de estos, 98 desarrollaron lesión renal aguda (80,3%). En la regresión múltiple, los factores asociados encontrados fueron el uso de vasopresina, el *Nursing Activities Score* y la tasa de filtración glomerular.

**Conclusión:**

El uso de vasopresina, el *Nursing Activities Score* y la tasa de filtración glomerular se consideraron factores relacionados con el desarrollo de lesión renal aguda en pacientes sometidos a oxigenación por membrana extracorpórea.

## INTRODUCTION

Extracorporeal membrane oxygenation (ECMO) is a highly complex temporary support indicated for patients with respiratory, cardiac or cardiorespiratory failure^(1)^. ECMO is related to the occurrence of renal dysfunctions since it frequently results in the need for renal replacement therapy (RRT), progression to chronic renal disease (CRD) and increased mortality^(2,3)^. Thus, continuous evaluation of the renal function of individuals undergoing ECMO is essential to avoid the development of complications and to prevent treatment in the critical phase of dysfunction from resulting in unfavorable outcomes^(2,3)^.

The Extracorporeal Life Support Organization (ELSO) reports that the in-hospital mortality of patients who used the support is 55%^(4)^. However, when the outcome is analyzed by the type of modality, the results are different: the veno-venous (VV) modality presents a mortality rate around 33% while the veno-arterial (VA) one reaches a mortality rate of 54%^(4)^.

Among the clinical factors that may contribute to an unfavorable outcome in critically ill patients, acute kidney injury (AKI) is worth of note^(5)^. The incidence of AKI in patients submitted to ECMO varies from 30 to 100%, and this rate differs according to the support modality employed, as previously mentioned when the mortality outcome was approached^(6)^. According to the type of access, the occurrence of AKI in patients with VV ECMO is 17%, and VA ECMO is 23%. When analyzed together, regardless of the modality, AKI requiring RRT is close to 28%^(5)^. It is noteworthy that due to the pandemic of Coronavirus disease 2019 (CoVID-19)^(7)^, this extracorporeal support was employed as a therapy in situations of severe acute respiratory failure (ARF), and in this context, the occurrence of AKI is expressive, besides resulting in significant need for RRT^(8)^.

The pathophysiological mechanisms involved in the alteration of the renal function with the use of ECMO are complex, multifactorial and time-dependent on exposure^(9)^. The renal function of individuals submitted to ECMO is influenced by individual clinical history, severity, condition of renal hypoperfusion, hypoxia and use of nephrotoxic agents, besides aspects related to extracorporeal support such as hemodynamic, hormonal, inflammatory alterations and intercurrences with the circuit^(10)^. It is noteworthy that these factors may be present before the beginning of support, during and after its use, also considering that the support itself may induce but also prevent AKI^(9)^.

The literature is clear about the development of AKI as a potential complication in patients submitted to extracorporeal support^(6)^. However, studies involving adult patients on ECMO, which show the causes of AKI comprehensively, associating its development with inflammation markers, vasoactive drug dependence (VAD) scores, fluid overload and nursing workload are scarce^(6)^. Additionally, it is known that data on this therapy are identified in a fragmented manner in the country, being restricted to large urban centers^(4)^, which have specific material resources and specialized intellectual capital.

In this scenario of growing need for highly complex therapeutic support, especially ECMO, it is essential to develop studies analyzing the impact of this therapy on renal function and its repercussion on the multidisciplinary team’s care work. Moreover, investigations on this topic offer theoretical and practical subsidies for early detection and treatment of clinical complications, enabling the proposition and standardization of local therapeutic protocols to improve the quality of care related to the use of this support in order to reduce mortality and hospital costs. Therefore, the objective of this study was to identify factors associated with the development of AKI in patients who underwent ECMO.

## METHODS

### Study Type

This is a retrospective cohort study.

### Setting

It was carried out in an adult intensive care unit (ICU) of an extra-large private hospital, located in the southern zone of São Paulo. The institution has approximately 700 beds, the adult ICU has 40 beds and offers general clinical and surgical care.

### Population and Selection Criteria

The population encompassed all patients who used ECMO, admitted to the aforementioned ICU, including patients over 18 years old, from 2012 to 2021, undergoing ECMO. Exclusion criteria were the existence of missing or incomplete data in the medical record. Patients with non-dialytic chronic renal disease (CRD) were included.

### Data Collection

Data from patients undergoing ECMO in the proposed collection period were already anonymized in the Research Electronic Data Capture (REDCap)®^(11)^ platform. Therefore, after approval of the study by the ethics committee, data extraction was performed, already anonymized, i.e., without variables that could identify or expose patients.

Thus, the variables extracted from the REDCap® platform were the clinical and demographic characteristics as follows: gender, age, weight, clinical history, ICU and hospitalization time, mechanical ventilation (MV) time, type of VAD, Simplified Acute Physiology Score 3 (SAPS 3), Sequential Organ Failure Assessment (SOFA), Vasoactive Inotropic Score (VIS), Neutrophil Lymphocyte Ratio (NLR) and the Nursing Activities Score (NAS) of the ICU admission; ECMO-related as ECMO indication diagnosis, modality, cannulation site, ECMO time, time between ICU admission and start of ECMO, Respiratory Extracorporeal Membrane Oxygenation Survival Prediction (RESP); Survival after Veno-Arterial ECMO (SAVE) and decannulation scores, and renal function by serum creatinine (SCr) and glomerular filtration rate (GFR) at hospital admission and hospital discharge/death^(6–8)^.

### Definitions

The VIS score defined the patients’ dependence degree on vasopressor and inotropic drugs and respective doses, immediately before the ECMO start^(12)^. Computation of this score was based on the doses of dopamine, dobutamine, milrinone, adrenaline, noradrenaline and vasopressin^(12)^.

The NLR was used because it is considered a marker of inflammation, calculated from the absolute values of neutrophils and lymphocytes, since ECMO, as an extracorporeal therapy, induces inflammatory response^(13)^. In this study, the NLR was calculated 72 hours after the start of ECMO.

Regarding AKI during the use of ECMO, we used the definition according to Kidney Disease Improving Global Outcomes (KDIGO) that classifies AKI into 3 stages^(14)^: patients with an increase of SCr ≥ 0.3mg/dL or between 1.5 – 1.9 times compared to baseline SCr were classified as stage 1; those with an increase of SCr between 2 – 2.9 times were classified as stage 2 and those with an increase of SCr ≥ 3 times were classified as stage 3^(14)^. It is worth noting that baseline SCr was considered as the first one collected on arrival on hospital admission.

The fluid overload was calculated by the sum of the fluid balance (FB) from the first to the fifth day of ICU stay, for patients who did not require RRT^(15)^. For those who required RRT, the FB sum was calculated from the day of ICU admission until the start of RRT^(15)^. Once the FB sum was obtained, calculation of fluid overload consisted of dividing the sum of the FB by the patient’s weight at hospital admission multiplied by 100^(15)^. Patients who presented values > 0.1 in this ratio, i.e., larger than 10%^(15)^ were considered to have fluid overload.

### Data Analysis and Treatment

The anonymized data from the REDCap®^(11)^ platform were exported using a Microsoft Excel® 2007 spreadsheet. For the analyses the software Statistical Package for the Social Sciences (SPSS)® for Windows® version 26.0 was used.

At a first step it was studied the relationship between the study variables and AKI a descriptive analysis of quantitative variables, based on the distribution type presented by the Shapiro-Wilk test, shown as median and 25% and 75% interquartile ranges. The qualitative variables were described by absolute and relative frequencies. In the inferential analysis, Fisher’s exact and chi-square tests were used for qualitative variables, and for quantitative variables the Mann-Whitney test was used.

For the purposes of the analysis of factors associated with AKI, a multiple logistic regression model was developed. First, variables with p < 0.2 were chosen to compose the simple logistic regression model in the analysis of the groups with and without AKI. Then, to compose the multivariate regression model, the variables with *p*≤ 0.05 found in the analysis of the simple logistic regression model were selected. The estimation of the Odds Ratio (OR) with respective 95% Confidence Interval (95% CI) was also evaluated, by means of both simple and multivariate logistic model. The Variance Inflation Factor (VIF) was used to test for collinearity. The significance level adopted for the analyses was 5%.

### Ethical Aspects

The study complied with the regulations of Resolution 466/2012 and was approved by the Research Ethics Committee of Hospital Israelita Albert Einstein, in 2022, according to opinion number 5,235,665.

## RESULTS

The study sample consisted of 122 individuals, since none was excluded for missing data. No patient at admission had acute renal function impairment. The prevalence of AKI was 98 patients (80.3%). Table 1 describes the demographic and clinical characteristics of patients with and without AKI. The median age was 55.0 years, with a higher prevalence of male patients (68.0%). The group with AKI had longer ICU hospital stay and use of MV, however, no statistical difference was confirmed. Noradrenaline stood out as the most used VAD (99.2%), and it is noteworthy that all patients underwent MV. The NFL marker and NAS were higher in the group with AKI (*p*< 0.05).

**Table 1.** Relationship between demographic and clinical characteristics of patients with and without AKI – São Paulo, SP, Brazil, 2021.

Variables	Total (n = 122)	No AKI (n = 24)	AKI (n = 98)	*p-value*
**Sex** Men	83 (68.0)	14 (58.3)	69 (70.4)	0.256*
**Age (years)**	55.0 (36.0–62.0)	48.5 (43.5–59.0)	56.0 (36.7–62.2)	0.180**
**Comorbidities**				
SAH	43 (35.2)	7 (29.2)	36 (36.7)	0.487*
CF	28 (23.0)	3 (12.5)	25 (25.5)	0.174*
DM	24 (19.7)	4 (16.7)	20 (20.4)	0.782
COPD	19 (15.6)	4 (16.7)	15 (15.3)	>0.999*
Cancer	12 (9.8)	1 (4.2)	11 (11.2)	0.457*
CRD	7 (5.7)	–	7 (7.1)	0.343***
Stroke	2 (1.6)	–	2 (2.0)	>0.999***
**CoVID-19**	44 (36.1)	5 (20.8)	39 (39.8)	0.083*
**Hospital LOS** (days)	31.5 (16.2–59.2)	24.0 (12.2–46.0)	32.5 (17.7–63.0)	0.208**
**ICU** (days)	21.5 (9.0–41.0)	13.5 (6.2–38.2)	24.0 (10.0–41.7)	0.172**
**MV** (days)	14.5 (5.0–33.2)	8.5 (4.2–38.2)	15.0 (5.0–33.2)	0.474**
**VAD Type**				
Noradrenaline	121 (99.2)	24 (100.0)	97 (99.0)	>0.999*
Adrenaline	67 (54.9)	11 (45.8)	56 (54.9)	0.318*
Dobutamine	52 (42.6)	10 (41.7)	42 (42.9)	0.916*
Vasopressin	32 (26.2)	2 (8.3)	30 (30.6)	0.026*
Milrinone	18 (14.8)	2 (8.3)	16 (16.3)	0.522*
**IAB**	26 (21.3)	4 (16.7)	22 (22.4)	0.535*
**Weight** (Kg)	78.0 (35.0–89.5)	73.0 (66.3–89.9)	78.5 (65.0–87.6)	0.978**
**SAPS 3**	48.5 (38.0–57.2)	51.0 (39.5–62.7)	47.0 (38.0–57.0)	0.285**
**SOFA**	9 (7.0–12.0)	8.5 (6.2–10.0)	9.5 (7.0–13.0)	0.178**
**VIS**	28.5 (9.7–132.5)	28.5 (10.0–143.8)	27.7 (2.5–93.1)	0.308**
**NLR**	10.2 (6.6–20.1)	11.0 (7.1–21.1)	6.4 (4.4–10.7)	0.001**
**NAS**	110.7 (99.3–122.0)	96.5 (89.0–99.7)	117.0 (105.3–124.0)	<0.001**
**Death**	76 (62.3)	12 (50.0)	64 (65.3)	0.166*

AKI: Acute Kidney Injury; SAH: Systemic arterial hypertension; CF: Cardiac failure; COPD: Chronic Obstructive Pulmonary Disease; DM: Diabetes Mellitus; CRD: Chronic Renal Disease; CoVID-19: *coronavirus disease 2019*; LOS: Length of stay; ICU: Intensive Care Unit; MV Mechanical Ventilation; IAB Intra-Aortic Balloon; Kg: Kilogram; SAPS: simplified acute physiology score; SOFA: sequential organ failure assessment score; VIS: vasoactive inotropic score; NLR: neutrophil lymphocyte ratio; NAS: Nursing activities score. Results are expressed by n (%), median (interquartile interval) or mean ± standard deviation. *: Chi-Square Test; **: *Mann-Whitney* Test; ***: Fisher ‘s Exact Test.

The variables related to the use of ECMO are described in Table 2. The most prevalent diagnosis for ECMO indication was acute respiratory failure (55.7%) and the VV modality was used in 55.7% of cases. Regarding cannulation of support, the peripheral type stands out, being observed in 89.3% of patients. The time between ICU admission and ECMO start was higher for patients with AKI (2.0 versus 0.0 days, *p*= 0.042).

**Table 2.** Relationship between the characteristics related to the use of ECMO – São Paulo, SP, Brazil, 2021.

Variables	Total (n = 122)	No AKI (n = 24)	AKI (n = 98)	p-value
**Indications for ECMO**				
ARF	68 (55.7)	12 (50.0)	56 (57.1)	0.528*
Cardiogenic shock	29 (23.8)	7 (29.2)	22 (22.4)	0.488*
ECPR	12 (9.8)	3 (12.5)	9 (9.2)	0.702**
Difficulty weaning from ECC	11 (9.0)	2 (8.3)	9 (9.2)	>0.999**
**Modality**				
VA	55 (45.1)	10 (41.7)	45 (45.9)	0.708*
VV	68 (55.7)	14 (58.3)	54 (55.1)	0.775*
**Canulation**				
Peripherical	109 (89.3)	24 (100.0)	85 (86.7)	0.070*
Central	14 (11.5)	–	14 (14.3)	0.070**
**Time in ECMO** (days)	7.0 (3.0–18.0)	4.5 (2.2-14.5)	8.0 (4.0–18.0)	0.129***
**Time elapsed from ICU and start of ECMO** (days)	1.5 (0.0–7.2)	0.0 (0.0-3.5)	2.0 (0.0–8.0)	0.042***
**RESP**	−2.5 (−4.0–0.0)	−2.0 (−3.0–1.0)	−3.0 (−4.0–1.0)	0.119***
**SAVE**	0.0 (−3.2–2.2)	2.0 (−1.0–4.0)	−1.0 (−4.0–2.0)	0.081***
**Decannulation**	68 (55.7)	16 (66.7)	52 (53.1)	0.229*

AKI: Acute Kidney Injury; ARF: Acute Respiratory Failure; ECPR: *extracorporeal cardiopulmonar resuscitation;* ECC: Extracorporeal Circulation; VV: veno-venous; VA: veno-arterial; ECMO: Extracorporeal membrane oxygenation; ICU: Intensive Care Unit; RESP: *Respiratory Extracorporeal Membrane Oxygenation Survival Prediction*; SAVE: *Survival after Veno-Arterial ECMO*. Results are expressed by n (%), median (interquartile interval) or mean ± standard deviation. *: Chi-Square Test; **: *Mann-Whitney* Test; ***: Fisher ‘s Exact Test.

Table 3 describes the renal function and FB characteristics of patients with and without AKI. The fluid overload of the AKI patients was significant when compared to the patients without AKI (41.8% versus 12.5%, *p*= 0.007). The FB was higher in the AKI group (3320.0 ml versus 861.6 ml, *p*= 0.030). Regarding RRT, we observed an overall rate of 65.6%, and regarding the type of RRT 15.6% used the intermittent method and 62.3% the continuous method.

**Table 3.** Relationship between renal function and FB of patients with and without AKI – São Paulo, SP, Brazil, 2021.

Variables	Total (n = 122)	No AKI (n = 24)	AKI (n = 98)	p-value
**SCr at admission** (mg/dl)	1.0 (0.8–1.5)	0.9 (0.6–1.1)	1.1 (0.8–1.7)	0.041*
**SCr at outcome** (mg/dl)	1.1 (0.7–1.9)	0.9 (0.5–1.2)	1.2 (0.8–2.0)	0.025*
**GFR at admission** (ml/min)	81.5 (48.4–104.6)	96.9 (72.0–120.5)	74.9 (44.7–99.5)	0.022*
**GFR at outcome** (ml/min)	70.7 (40.0–111.5)	103.4 (56.2–139.7)	63.0 (39.6–99.7)	0.012*
**FB** (ml)	3230.5 (237.5–6787.5)	861.6 (–2249.1–2778.5)	3320.0 (525.0–7022.5)	0.030*
**Fluid Overload**	44 (36.1)	3 (12.5)	41 (41.8)	0.007**

AKI: Acute Kidney Injury; Cr: Serum creatinine; GFR: glomerular filtration rate; FB: Fluid Balance; ICU: Intensive Care Unit; ECMO: Extracorporeal membrane oxygenation. Results are expressed by n (%), median (interquartile interval). *: Chi-Square Test; **: *Mann-Whitney* Test.

**Figure 1.**
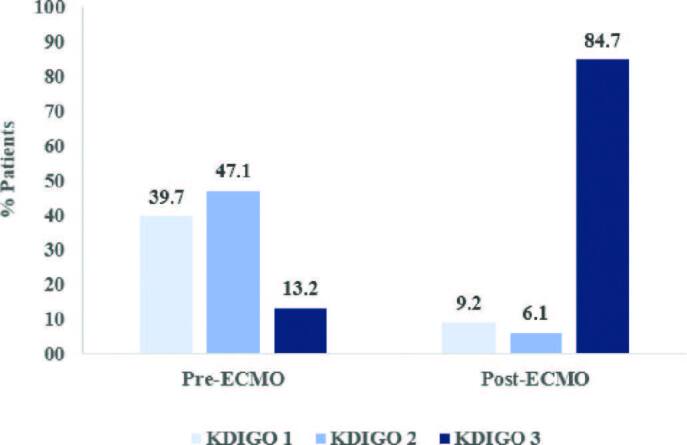
Comparison of AKI classification by KDIGO between before and after the start of ECMO – São Paulo, SP, Brazil, 2021.

AKI: Acute Kidney Injury; KDIGO: *Kidney Disease Improving Global Outcomes;* ECMO: Extracorporeal membrane oxygenation.

Figure 1 shows the AKI classification by KDIGO between the periods before and after the start of ECMO. It can be observed that the most prevalent classification before support was stage 2, however, after the start of ECMO stage 3 was the most identified.

Table 4 shows the univariate and multivariate analyses referring to the multiple logistic regression model for factors associated with AKI. In the univariate analysis, the variables considered to have statistical significance were vasopressin use (OR: 4.85; 95%CI: 1.07–21.96; *p*= 0.040), NAS (OR: 3.33; 95%CI: 1.38–8.04; *p*< 0.001) and GFR on admission (OR: 0.98; 95%CI: 0.94–0.99; with *p*= 0.023).

**Table 4.** Univariate and multivariate analyses referring to the multiple logistic regression model for factors associated with AKI – São Paulo, SP, Brazil, 2021.

	Univariate			Multivariate		
	OR	CI 95%	p-value	OR	CI 95%	p-value
**Vasopressin**	4.85	(1.07–21.96)	0.040	11.41	(1.21-10.86)	0.033
**NAS**	3.33	(1.38–8.04)	<0.001	1.24	(1.11-1.38)	<0.001
**CoVID-19**	2.51	(0.86–7.28)	0.090	–	–	–
**CF**	2.39	(0.65–8.72)	0.185	–	–	–
**SOFA**	1.08	(0.96–1.21)	0.189	–	–	–
**Time elapsed from ICU and start of ECMO** (days)	1.08	(0.98–1.19)	0.095	–	–	–
**NLR**	1.05	(0.99–1.11)	0.087	–	–	–
**Time in ECMO** (days)	1.01	(0.98–1.04)	0.401	–	–	–
**Age**	1.01	(0.98–1.04)	0.267	–	–	–
**GFR at admission**	0.98	(0.94–0.99)	0.023	0.97	(0.95-0.99)	0.014

OR: Odds Ratio, 95% CI: 95% confidence interval; NAS: Nursing activities score, CoVID-19: coronavirus disease 2019; CF: cardiac failure; SOFA: sequential organ failure assessment score; ICU: intensive care unit; ECMO: extracorporeal membrane oxygenation; NLR: neutrophil lymphocyte ratio, GFR: glomerular filtration rate.

Regarding multiple analysis, the factors associated with the development of AKI were vasopressin use (OR: 11.41; 95%CI: 1.21-10.86; with *p*= 0.033), ICU admission NAS (OR: 1.24; 95%CI: 1.11–1.38; with *p*< 0.001) and admission GFR (OR: 0.97; 95%CI: 0.95–0.99; with *p*= 0.014). It is worth noting that the VIF ranged from 1.01–1.02, thus confirming the absence of collinearity between variables.

## DISCUSSION

The increased use of ECMO in the last decade confirms the therapy as an alternative for treatment of critically ill patients. However, this trend requires better preparation of multidisciplinary teams, since the occurrence of clinical complications impacts on the outcome of these individuals^(2,4)^. Among the complications, the present study demonstrated that the renal function of patients submitted to ECMO changes, resulting in a significant prevalence of AKI, as already demonstrated in a previous study^(16)^.

AKI related to the use of ECMO has multifactorial causes such as factors prior to the start of ECMO, highlighting comorbidities, use of nephrotoxic agents and the state of systemic inflammation, in addition to factors detected after the beginning of therapy, such as changes in the blood flow, hormonal agents, inflammation resulting from the contact of blood with the circuit and hemolysis^(10)^.

Flow alterations with impact on hemodynamics, a relevant factor as complication of the therapy, are causes of AKI, since a prolonged period of hypotension associated with low tissue perfusion are predisposing conditions for reduced renal perfusion^(10)^. It has already been described that in non- cardiac surgeries in which the mean arterial pressure (MAP) is maintained below 55 mmHg for a period longer than 40 minutes, the risk of renal function alteration increases fourteen times, and this condition resembles the hemodynamic context that the patient is exposed when using ECMO^(17)^. Additionally, it was observed that critically ill patients with stage 1 AKI, if exposed to MAP levels lower than 65 mmHg for more than one hour, present a risk of worsening of the renal function severity, with progression to stage 3^(18)^. This fact was also observed in the present study, since the evolution of AKI, from initial stages 1 and 2 before support, to stage 3 after the installation of therapy.

The use of nephrotoxic drugs is also translated as risk factors for AKI in patients on ECMO. In this scenario, the use of vasopressor drugs may have a hemodynamic rescue effect on the critically ill patient, but it is also a nephrotoxic agent, since it can induce tissue hypoperfusion and compromise renal hemodynamics^(19)^. For this reason, vasopressors are considered as predictors for AKI, for increasing twice the risk, besides being related to the indication of RRT for prolonged periods^(19)^. The association of more than one vasopressor agent or the administration of high doses increases the risk of AKI^(20)^. In this study, the relationship between the use of vasopressors, such as vasopressin, and its association with noradrenaline and AKI was confirmed.

In this sense, the VIS score is a relevant tool to estimate the dose of vasopressors and inotropic agents, and to relate this score with possible clinical outcomes^(12)^. A recent study on the relationship between the VIS score and the occurrence of AKI after cardiac surgery showed a positive prediction and the score was considered an independent predictor of AKI^(12)^. Although the VIS score was higher in patients with AKI in the present study, it was not confirmed as a factor associated with AKI.

On the other hand, the GFR on hospital admission was related to the occurrence of AKI. However, even not being included in the KDIGO criteria for AKI, the GFR values are important to help in detection, understanding the severity, decision about diagnosis, prognosis and treatment of renal dysfunction^(21)^. Conditions such as AKI or acute CKD present with reduced baseline GFR, i.e., higher GFR values are related as a protective factor for renal function, which is in agreement with the present study^(22)^.

It was observed that reduced baseline GFR values at hospital admission in the perioperative period is shown as a risk factor for the development of AKI and progression in CRD^(23)^. The AKI staging stratifies the severity of renal impairment in such a way that the evolution from stage 1 to stage represents the reduction of GFR^(23)^. Among patients undergoing ECMO, it was observed that the GFR from admission up to the first 30 days of hospitalization gradually increases for those who evolve without AKI, however, for those with AKI, there is a decrease in the GFR on admission over time, especially in relation to stage 3^(24,25,26)^.

This study also demonstrated that the nursing workload, estimated by the ICU admission NAS, was confirmed as a factor associated with the occurrence of AKI. To the best of our knowledge, this is the first Brazilian study to confirm NAS as a factor associated with AKI in patients on ECMO. The discriminatory power of NAS is known to indicate the high demand of care by the nursing team^(27)^, besides that the importance of the nurse’s performance in the context of extracorporeal support, with characteristics of high complexity and need of advanced care^(28)^ is unquestionable, including from the careful clinical evaluation of the patient to the monitoring of the system to guarantee a safe practice^(27)^.

In addition to patient evaluation and system monitoring, nursing care in a situation of imminent risk of AKI should include control of laboratory tests, pressure control, control of urine output and FB, which should be intensified in the presence of RRT^(29)^.

These data reinforce the need to assure that the staffing ratio of nurses is 1:1, that is, one professional nurse for the direct care of a patient submitted to ECMO, in order to assure the patient’s and professional’s safety^(27)^. The statement made by the Sao Paulo Regional Nursing Council (COREN-SP) under opinion 033/2011 that emphasizes the role of nurses as the person responsible for direct care of patients undergoing ECMO is also noteworthy^(30)^. However, currently in Brazil, unfortunately, these staffing proportions are restricted to large hospital centers.

In view of the above, the study confirms that the use of ECMO and the clinical conditions of patients who need this therapy are factors associated with the occurrence of AKI and that the nurse’s performance and his/her dialogue with the multidisciplinary health team is fundamental in the occurrence of clinical complications. However, this study was limited by the fact that it was uni-centered, pointing to the need for multicenter research in order to provide evidence to support assertive decision making by multidisciplinary teams.

## CONCLUSIONS

The factors associated with the development of AKI were the use of vasopressin, the NAS at ICU admission, and the GFR at hospital admission. Thus, actions such as careful hemodynamic monitoring, the performance of a qualified nursing team for highly complex care, with an adequate proportion of nurses, and the evaluation of renal function markers at the time of hospital admission are fundamental elements to avoid the occurrence of AKI.
